# Visual and Surgical Outcomes of Outreach Cataract Surgeries in Ilam District of Nepal: An Observational Study

**DOI:** 10.31729/jnma.8798

**Published:** 2024-11-30

**Authors:** Pragya Luitel, Manish Pandey, Rajiv Ranjan Karn, Mahesh Kumar Dev, Lily Rajbanshi, Rakshya Panta Sitoula

**Affiliations:** 1 Cornea Department, Biratnagar Eye Hospital, Biratnagar, Morang, Nepal; 2 Research Department, Biratnagar Eye Hospital, Biratnagar, Morang, Nepal; 3 Retina Department, Biratnagar Eye Hospital, Biratnagar, Morang, Nepal; 4 Glaucoma Department, Biratnagar Eye Hospital, Biratnagar, Morang, Nepal

**Keywords:** *cataract*, *complications*, *eye camp*, *outreach*, *visual acuity*

## Abstract

**Introduction:**

Cataracts are the leading cause of preventable blindness worldwide. Although cataract blindness is reversible, its service coverage remains poor, particularly in rural and hilly areas of Nepal. The study aimed to evaluate visual outcomes of outreach cataract surgeries and associated intraoperative and post-operative complications.

**Methods:**

This cross-sectional study was conducted at a district of Eastern Nepal after ethical clearance was taken from the Institutional Review Committee (Reference number: 88). Total 131 subjects had cataract surgeries at the surgical camp, and their pre-operative, one-day and one-month post-operative visual acuities were compared, and intraoperative and post-operative surgical complications were recorded. Visual acuity ≥ 6/12 was considered normal. Data was collected in excel and analyzed in SPSS.

**Results:**

The mean age of participants was 71.8 ± 9.51 years, with 70 (53.43%) male. A total of 156 eyes from 131 subjects underwent cataract surgeries and among them 25 (19.08%) cases underwent bilateral cataract surgeries. At the day one of surgery, visual acuity improved to normal in 137 (87.82%) of cases, while after one-month, visual acuity improved to normal in 150 (96.15%) of cases with best-corrected glasses. There were 4 (2.56%) intraoperative complications, which included surgery-induced subluxated bag, premature entry, iris trauma, and posterior capsule rent.

**Conclusions:**

The visual and surgical outcomes of cataract surgeries performed at an outreach surgical camp were excellent with minimal surgical complications.

## INTRODUCTION

Globally, 2.2 billion people have vision impairment, and almost half of this impairment could be prevented or treated.^1^ Uncorrected refractive error and cataract are the largest contributors to avoidable visual impairment and blindness in adults aged 50 years and older. The prevalence of vision impairment in low-and middle-income countries is estimated to be four times higher than in high-income regions.^2^

An estimated 8 million people in Nepal need eye care services annually, however, only 1.5 million accessed services in 2010.^3^ Cataract is the leading cause of preventable blindness in Nepal,^4^ mostly in rural and hilly regions.^5^ Addressing the increasing backlog of cataracts is challenging. To combat these backlogs, one of the effective methods is outreach surgical camps.^6^ There are limited studies that have evaluated the visual outcomes of cataract surgeries after one month in Eastern Nepal. This study aimed to compare cataracts pre and post-operative visual acuity and determine associated surgical complications.

## METHODS

This was an observational, cross-section study conducted among 1643 participants recruited from an outreach surgical camp held at Fikkal, Suryodaya Municipality, Ilam, which is located in province one of Nepal between the 16^th^ to 18^th^ of September 2022. Prior to the surgical camps, Diagnostic, Screening, and Treatment (DST) camps were held in all 14 wards of Suryodaya Municipality. Ethical approval was taken from the municipality before the program and ethical clearance was taken from the Institutional Review Committee (IRC) of Biratnagar Eye Hospital (Reference number: 88). This study followed the tenets of the Declarations of Helsinki. The information of stipulated date of surgical camps was circulated using audio-visual means such as local radio or pamphlets. Experienced ophthalmic assistants from Primary Eye Care Centers (PECC) screened all the patients from Fikkal and nearby places who visited DST camps. They tested vision and conducted cataract screening using a simple torch light. All patients who were screened to have cataracts of any severity were allocated a specific date and asked to follow-up in the PECC for a detailed eye examination by an ophthalmic surgeon.

At the PECC, a surgical team of 7 ophthalmic personnel (1 ophthalmic surgeon, 1 operation theatre supervisor, 1 ophthalmic assistant, 2 eye health workers, and 2 nurses) from Biratnagar Eye Hospital (a tertiary eye care hospital) conducted further eye examination and surgical procedures. All cataract-screened patients underwent a comprehensive eye examination again on the day of surgery by an experienced ophthalmic surgeon. A complete eye examination was done: Visual acuity was tested monocularly with a Snellen chart under normal room illumination, anterior segment examination was done with a slit lamp and dilated posterior segment examination was carried out by either direct ophthalmoscope or 90 Diopter lens. The patients who were diagnosed with cataracts underwent surgery on the same day, while those associated with other eye diseases such as age-related macular degeneration, glaucoma, or any other retinal diseases were referred to Biratnagar Eye Hospital for further evaluation and management. Those who were diagnosed with refractive error were prescribed glasses after refraction.

For cataract surgery, two rooms of a local primary school were identified. Thorough sterilization was done in the surgery and block rooms, simulating the operation theatre of hospital settings. The best sterilization protocol was applied that was feasible in outreach settings. Fumigation was done with Aldehyde free fumigant (Dr. OT-141).

All investigations including Intraocular pressure, Blood pressure, Random Blood Sugar (Glucometer) and Biometry were carried out by ophthalmic assistants. All patients were explained about the nature and scope of this surgery and written consents were obtained from them.

Before surgery, visual acuity, demographics (age, gender, and ethnicity classified according to the Central Bureau of Statistics of Nepal) intra-ocular lens (IOL) power and Operating Eye (Left or Right eye) were noted and confirmed and then a retrobulbar block or peribulbar block was given in the eyes to be operated in the block room and then proceeded for surgery. Manual Small Incision Cataract Surgeries (SICSs) with intraocular lens implantations were performed in all patients with cataracts. The SICS is sutureless surgery where the whole nucleus is removed through a self-sealing sclero-corneal tunnel.^7^ Capsulorrrhexis was done in every cases. Trypan blue was used when needed. A single-piece Poly Methyl Methacrylate IOL was inserted into the capsular bag. Intracameral cefuroxime (0.1 mL of 10.0 mg/ mL) was injected after the formation of anterior chamber. Sub conjunctival injection of dexamethasone (2 mg) and gentamicin (20 mg) was given at the end of the procedure. The sclerocorneal tunnel was self-sealed and suture was not placed. This technique requires no sophisticated equipment nor any special machine, sutureless, provides quick rehabilitation and gives an excellent outcome.^8^ In addition to cataract surgeries, some other minor extraocular surgeries were also done in some of the patients.

After cataract surgery, patients were kept for 1 day at the PECC for observation, and on the next day, operated eyes were examined and provided with the post-operative eye drops (combination of Ofloxacin 0.3% and Dexamethasone Sodium Phosphate 0.1%) on tapering dose and were asked to follow-up after a month at PECC. The outcomes variables were visual acuity and intra- and post-operative surgical complications. Uncorrected visual acuities were tested in the operated eyes at one-day and after one-month post-operation, following similar testing procedures at the baseline visual acuity. Moreover, at one-month, objective and subjective refractions were performed by senior ophthalmic assistant to determine the best corrected visual acuities in the operated eyes. Visual acuities were classified using the International Classification of Diseases 11 (2018): Distance vision impairment: Mild - presenting visual acuity worse than 6/12, Moderate - presenting visual acuity worse than 6/18, Severe - presenting visual acuity worse than 6/60, Blindness - presenting visual acuity worse than 3/60.^1^ All cataract complications that occurred during intraoperative as well as post-operative were also recorded. The preoperative, intraoperative and postoperative data were recorded in prescription card manually by eye health worker or ophthalmic assistant.

The data were entered in excel sheet by outreach coordinator and kept in hospital records. Statistical analyses were performed using SPSS statistics 20.0 (IBM, Armonk, NY; ibm.com). The descriptive analysis was done, where for continuous data, means and standard deviations (SDs) were calculated, while for categorical data, frequencies and percentages were calculated.

## RESULTS

A total of 156 eyes of 131 patients underwent cataract surgeries and out of them, 25 (19.08%) patients underwent surgeries in both eyes. Surgery in the left eye was done in 78 (50%) patients and 78 (50%) patients in the right eye. The mean age of patients was 71.8±9.51 years and there were 70 (53.43%) male who underwent the surgery.

The power of IOL implanted ranged from 16.0 to 29.0 D. Uncorrected visual acuity improved to normal in 104 (66.66%) of patients on the day of post-operation. After one month of post-operation, uncorrected visual acuities improved to normal in 137 (87.82%) and best corrected visual acuities improved to normal in 150 (96.15%) of patients (Table 1).

**Table 1:** Comparison of pre-operative, one-day and one-month post-operative visual acuity of 131 patients who underwent cataract surgeries (n=156).

Categories of visual impairment	Pre-operative UCVA, n(%)	1 day Post-operative UCVA, n(%)	1 Month Post-operative UCVA, n(%)	1 Month Post-operative BCVA, n(%)
Normal	6 (3.84)	104 (66.66)	137(87.82)	150 (96.15)
Mild visual impairment	40 (25.64)	37 (23.71)	11(7.05)	1 (0.64)
Moderate visual impairment	54 (34.61)	13 (8.33)	4(2.56)	1 (0.64)
Severe visual impairment	34 (21.79.1)	-	-	-
Blindness	22 (14.10)	2 (1.28)	-	-
Missed follow-up	-	-	4 (2.56)	4 (2.56)
Total	156	156	156	156

UCVA = Uncorrected Visual Acuity, BCVA = Best corrected visual acuity

There were 4 (2.56%) intra-operative and 4 (2.56%) postoperative complications. Intra-operative complications included subluxated bag 1 (0.64%), premature entry 1 (0.64%), iris trauma 1 (0.64%), and posterior capsule rent 1 (0.64%); post-operative complications included corneal oedema in 2 (1.28%) cases and hyphema in 1 (0.64%), and air in anterior chamber in 1 (0.64%) case. Anterior chamber wash was done for hyphema. Corneal edema and air in anterior chamber subsided on follow up.

## DISCUSSION

Among the 156 operated eyes of 131 patients, visual acuity improved to normal in 137 (87.82%) of cases on the day one of surgery, while after one-month, visual acuity improved to normal in 150 (96.15%) of cases with best-corrected glasses. Importantly, only 2.6% intraoperative complications. Therefore, outcome of cataract surgeries was excellent with minimal surgical complications and excellent visual outcome in the outreach surgical eye camp. This highlights the need for outreach cataract surgeries in rural community settings where access to eye care services are not easily available or affordable.

The mean age of patients was 71.8±9.51 years in this study. patients seeking for cataract surgery were of similar age range in the current study, and previous studies conducted by Rasaily S and Kharel SR,^6,7^ indicating that similar cohort of people seek eye care services in rural area of Nepal. In general females and marginalized communities used to get more benefit from free surgical eye camps.^6,9^ The prevalence of blindness is also high among females in Nepal.^10^ There were more male participants in this study group as possibly more male were screened at the time of study. Though cataract was seen more in female, there was no significant association between gender and cataract.^12^ Likewise, here the major portion of participants were from Janajati community i.e 55.72%, similar to the study done by Rasaily et al. where marginalized community were benefited from surgical camp.^6^

In some of the studies^7,9^, visual outcome is measured only on first post operative day. We have taken visual acuity on first as well one month post operative day. In our study at first post operative day, visual acuity improved to normal 66.66% using International Classification of Diseases (ICD-11) . Importantly, the best corrected visual acuity improved to normal by 96.15% after 1 month which is similar to the study done by Gurung R et al., where the best corrected visual acuity improved to 93.2%^11^ and the study done by Nowak R et al, where this improved to 98.6%.^12^ The reason for not improving best corrected visual acuity to normal in all patients was due to residual refractive error, astigmatism, hypertensive and diabetic retinopathy. . The risk factors of worse visual outcome of cataract surgeries may include age related macular degeneration, diabetic retinopathy, corneal pathology, older age, previous vitrectomy, previous retinal and choroidal detachment, and intraoperative complications.^13^

In this study, 2.56% of both intra- and post-operative complications were reported, which is consistent with the previous study, which showed 1.2% complications at Diktel,.^12^ However, higher surgical complications were noted in other studies 6.7% ^11^ and 6% in Dhading.^7^ These variations in the surgical complications (Premature entry, Posterior Capsular Rent, Hyphema, Corneal edema) at outreach surgical camps may be due to surgeon factors, case selection, patients compliance with medicines and follow up.

It is important to note that cataract backlog still remains a public health problem, especially in rural and hilly areas of Nepal.^6^ There is a need for frequent surgical eye camps for people living in rural area and for those who are socially, economically, and politically disadvantaged and have less access to eye care services.^9^ The practice of surgical outreach to address cataract backlogs is a proactive approach adopted by many countries, recognizing the significant impact cataracts have on vision and quality of life.^14,15^

Outreach programs are an integral part of the Nepalese eye care delivery system. ^16^ The problem still exists in terms of the utilization of available services. Engaging local community leaders can significantly enhance patient participation in surgical eye camps in several ways - through trust building, awareness and education, cultural reassurance, and logistical support. With regards to this study, conducted in Suryodaya Municipality, which has been considered to have an educated people were still reluctant to come over to tertiary eye hospital in the Terai region. This might be due to the extremely hot climate at the Terai regions as well as due to the financial burden related to travel. The other reason might be that patients are more accustomed to surgical camps with high hope and faith.

The strength of this study is that we have taken the ICD-11 classification for visual impairment.-International Classification of Diseases 11 (2018): Distance vision impairment to measure the visual outcome.^1^ The findings of the study will provide baseline data for the visual outcome of outreach cataract surgeries. There are many limitations of this study, first one is intra-observer reliability and agreement which were not accessed for performing biometry and so there is likely to be some variation in IOL power calculations, which in terms affects visual acuity. Second, this includes only one camp data and findings may not be generalized. Therefore, this study warrants the need for future study with a larger sample size with different places of Eastern Nepal.

## CONCLUSIONS

The surgical outcomes of outreach cataract surgeries done in surgical camp were good, with minimal surgical complications and excellent visual outcomes. Most of the patient undergoing cataract surgery had normal visual acuity after one month of the surgery.

## References

[1] World Health Organization (2021). Blindness And Vision Impairment: WHO Factsheet..

[2] GBD 2019 Blindness and Vision Impairment Collaborators; Vision Loss Expert Group of the Global Burden of Disease Study. (2021). Causes of blindness and vision impairment in 2020 and trends over 30 years, and prevalence of avoidable blindness in relation to VISION 2020: the Right to Sight: an analysis for the Global Burden of Disease Study.. Lancet Glob Health..

[3] Gudlavalleti MV (2009). Vision 2020: The Meaning And The Spirit Of The Right To Sight Initiative.. Nepal J Ophthalmol..

[4] Thapa SS, Berg RV, Khanal S, Paudyal I, Pandey P, Maharjan N (2011). Prevalence Of Visual Impairment, Cataract Surgery And Awareness Of Cataract And Glaucoma In Bhaktapur District Of Nepal: The Bhaktapur Glaucoma Study.. BMC Ophthalmol.

[5] Manandhar LD, Rai SK, Bajracharya K, Kandel RP, Sharna P, Bassett K (2018). Cataract Surgical Quality And Cost In A Hill Region Of Western Nepal: Comparing Outreach Eye Camps With Base Hospital.. Asian J Med Sci..

[6] Rasaily S, Pokhrel K, Subedi S, Katuwal S, Gautam SOPY (2021). Access Of Marginalized Groups For Free Surgical Camp In Remote Areas Of The Midwestern Region Of Nepal.. J Community Med Public Heal Reports..

[7] Sitaula RK, Joshi SN, Khanal S (2020). Surgical Eye Camp In Rural Area Of Nepal And Its Role In Vision 2020.. J Chitwan Med Coll..

[8] Gurnani B, Kaur K (2023). StatPearls [Internet]..

[9] Gurung J, Tuladhar S, Sharma A (2019). The Role Of Eye Camps In Rural Areas Of Nepal. J Ganki Medical College Nepal..

[10] Brilliant LB, Pokhrel RP, Grasset NC, Lepkowski JM, Kolstad A, Hawks W (1985). Epidemiology Of Blindness In Nepal.. Bull World Health Organ..

[11] Nowak R, Grzybowski A (2013). Outcome Of An Outreach Microsurgical Project In Rural Nepal.. Saudi J Ophthalmol..

[12] Gurung R (2007). Cataract Surgical Outcome And Gender-Specific Barriers To Cataract Services In Tilganga Eye Centre And Its Outreach Microsurgical Eye Clinics In Nepal.. Community Eye Health..

[13] Gaskin GL, Pershing S, Cole TS, Shah NH (2016). Predictive modeling of risk factors and complications of cataract surgery.. Eur J Ophthalmol..

[14] Javaloy J, Signes-Soler I, Moya T, Litila S (2021). Cataract Surgery In Surgical Camps: Outcomes In A Rural Area Of Cameroon.. Int Ophthalmol..

[15] Senjan Suraj, Vashist Praveen, Malhotra Sumit (2014). Outcome of Cataract Surgery from Outreach Eye Camp.. Delhi Journal of Ophthalmology..

[16] Ruit S, Tabin G, Chang D, Bajracharya L, Kline DC, Richheimer W (2007). A Prospective Randomized Clinical Trial Of Phacoemulsification Vs Manual Sutureless Small-Incision Extracapsular Cataract Surgery In Nepal.. Am J Ophthalmol..

